# Effectiveness of Buzzy BEE in Reducing Pain Perception During Inferior Alveolar Nerve Block in Children: A Split-Mouth Crossover Study

**DOI:** 10.3390/children13060840

**Published:** 2026-06-22

**Authors:** Prabhadevi C. Maganur, Satish Vishwanathaiah, Renad Hussain Mohammed Ariji, Shaima Mansour Alabdali, Nawar Ebrahem Ahmed Moafa, Mohammed Jafer, Hammam Ahmed Bahammam, Noura Alessa, Ahtesham Ahmad Qurishi, Ahmed Ibrahim Atiah Ruwayni, Esraa Eissa Ibrahim Abujamilah, Bushra Mohammed Ahmad Wasili, Wejdan Faris Saleh Alhaider, Anas Ali Mohammed Dahmas

**Affiliations:** 1 Department of Preventive Dental Sciences Division of Pediatric Dentistry, College of Dentistry, Jazan University, Jazan 45142, Saudi Arabia; 2 College of Dentistry, Jazan University, Jazan 45142, Saudi Arabiaanasdhmesi67@gmail.com (A.A.M.D.); 3 Department of Preventive Dental Sciences Dental Public Health Division, College of Dentistry, Jazan University, Jazan 45142, Saudi Arabia; 4 Department of Pediatric Dentistry, Faculty of Dentistry, King Abdulaziz University, Jeddah 21589, Saudi Arabia; habahammam@kau.edu.sa; 5 Department of Pediatric Dentistry and Orthodontics, Dental College, King Saud University, Riyadh 11545, Saudi Arabia; nalessa@ksu.edu.sa; 6 Department of Maxillofacial Surgery and Diagnostic Sciences, College of Dentistry, Jazan University, Jazan 45142, Saudi Arabia; aqurishi@jazanu.edu.sa; 7 Jazan Health Cluster, Ministry of Health, Jazan 82722, Saudi Arabia; aruwayni@gmail.com (A.I.A.R.); dr.esraa.eissa@gmail.com (E.E.I.A.); wejdanalheder@gmail.com (W.F.S.A.); 8 Aseer Health Cluster, Ministry of Health, Al Rabwah, Abha 62523, Saudi Arabia; bushra.w1994@gmail.com

**Keywords:** Buzzy BEE, external vibration, inferior alveolar nerve block, pain perception, paediatric dentistry

## Abstract

Background: The paediatric dentistry domain requires effective management of pain in children during invasive procedures such as the inferior alveolar nerve block (IANB). This study aimed to evaluate the effectiveness of Buzzy BEE^TM^ in reducing pain perception during IANB in children. Methods: This crossover study recruited 38 children aged 6–10 years scheduled for bilateral dental procedures requiring IANB. The washout period was 7 days, and two visits were scheduled for procedures on either side. Two randomly allocated groups received the study intervention, with each group receiving it alternately at each visit. Outcomes measured were pulse rate; subjective pain assessment using the Wong–Baker FACES Rating Scale (WBS); objective pain assessment using the Sound, Eyes and Motor (SEM) scale; and parental rating of observed pain on a scale of 1–10. Results: A statistically significant reduction in pulse rate after IANB was observed in the Buzzy BEE group at the first (*p* = 0.02) and second (*p* = 0.002) visits. At the second visit, the WBS scores (*p* < 0.001) and ‘eye’ (*p* = 0.004) and ‘motor’ (*p* = 0.002) scores on the SEM scale were significantly reduced in the Buzzy BEE group. The crossover analysis identified a significant treatment effect on pulse rate (*p* < 0.001) and significant carryover effects on WBS and SEM scores (*p* < 0.001). Conclusions: The use of Buzzy BEE reduced pulse rates during IANB, suggesting a positive impact on children’s anxiety. The carryover effects in the current study limit the consistency of improvements in subjective and objective pain perceptions. Further studies with a larger sample size and an extended washout period are recommended to evaluate the effectiveness of Buzzy BEE in reducing pain perception during IANB.

## 1. Introduction

Childhood experiences of pain may influence an individual’s comprehension and perception of pain in adulthood [1]. Pain management in patients is a critical determinant of the outcome of dental treatments. The apprehension associated with dental procedures has led individuals to adopt a ‘wait-and-watch’ strategy, postponing or avoiding dental treatment whenever feasible [2]. Dental anaesthesia has consistently been essential for alleviating pain in patients [3]. Trypanophobia, or the overwhelming fear of needles or injections, is a leading cause of pain in patients with dental problems [4]. Approximately 12% of patients in juvenile dentistry have been shown to receive inadequate local anaesthesia (LA), which increases the need for additional analgesics and alters the anxiety response [5].

Thus, in paediatric dentistry, managing children’s pain effectively is not only essential but also challenging [6]. Family attitudes, facial trauma, heightened emotional sensitivity, low pain tolerance and prior unfavourable dental experiences are strongly associated with restricted cooperation owing to dread and nervousness [4]. In addition to these psychological factors, and physical difficulties—such as managing small oral structures and addressing growth-related changes in children’s teeth—can significantly impede the delivery of high-quality dental treatment [7]. Various strategies, including physical, pharmacological and psychological approaches, have been proposed to alleviate pain, such as pre-cooling the injection site, using topical anaesthetic gel, altering the infiltration speed, using intra-oral vibrating devices, using computer-controlled delivery systems and diverting attention [2]. Diverting attention from unpleasant stimuli while anaesthesia is being administered is a safe and effective behaviour-control technique that reduces pain and anxiety [4].

External cold and vibration devices have been used to distract paediatric patients during LA injections, attracting considerable attention in recent years [8,9,10,11]. Brain cells are thought to relay non-pain signals (e.g., cold or vibration) from the distracting environment created by the buzzing or vibrating device, obscuring the pain signals triggered by the injection [12]. The concealment of pain is due to confusion in pain pathways during signal perception [2,13].

The Buzzy BEE (MMJ Labs, Atlanta, GA, USA) is a non-invasive, reusable, low-cost, pain-relieving device shaped like a bee. This device provides a frigid sensory experience through detachable ice wings attached to a battery-powered vibrating unit. It was created by Dr Amy Baxter, a paediatric emergency physician practising in the United States [14]. Its design is grounded in Melzack and Wall’s 1965 gate control theory and the descending inhibitory mechanism [15]. During dental injections, this device has been shown to reduce discomfort [14,16,17], although most studies use infiltration as the anaesthetic [18,19,20,21]. Evidence regarding its efficacy in more invasive procedures, such as the inferior alveolar nerve block (IANB), remains unexplored. Only some studies have used the device for dental treatments requiring IANB anaesthesia [2,16,22,23]. Hence, this study was conducted to assess the credibility of Buzzy BEE in alleviating pain perception in children during IANB.

## 2. Materials and Methods

### 2.1. Study Design, Ethical Clearance and Informed Consent

**Figure 1:**
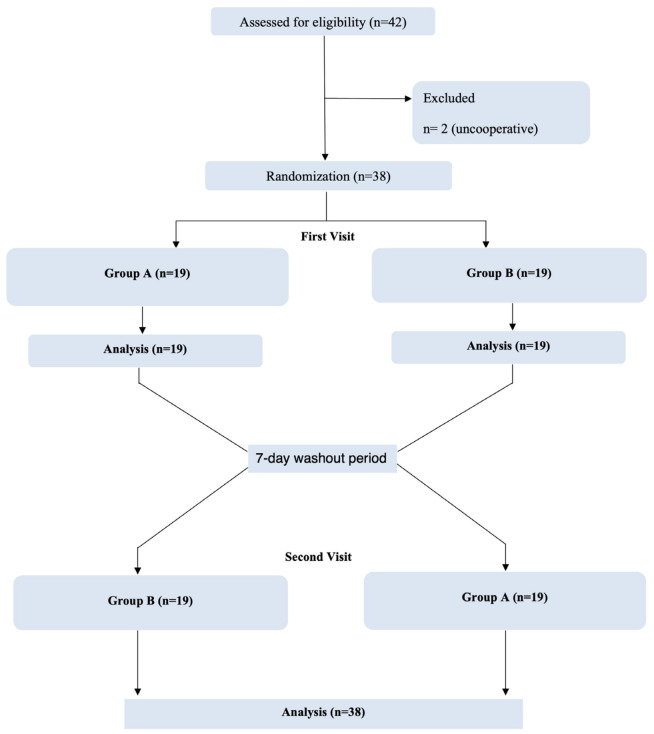
Consolidated flow chart.

A split-mouth crossover study was conducted in accordance with the Consolidated Standards of Reporting Trials (CONSORT) 2010 statement: extension to randomised crossover trials to ensure transparent reporting [13]. The CONSORT diagram (Figure 1) outlines the study participants. The local committee for research ethics at Jazan University (HAPO-10-Z-001) granted ethical clearance for the study (REC-47/08/1763). The study was registered in the national clinical trial registry, reference no. NCT07324915. Written informed consent from the participants’ parents and assent from the children were obtained before the trial commenced. Prior to enrolment, the parents or legal guardians were informed about the trial’s comprehensive protocol. The ethical guidelines of the 1964 Declaration of Helsinki and its most recent amendments were followed during the trial.

### 2.2. Estimation of the Sample Size

The sample size was estimated using the group parameters from a previous study [24]. The formula used to estimate the sample size is given below:

$$\mathrm{Sample}\;\mathrm{Size}\;(n)=\frac{2S_{p}^{2}{\left(Z_{1-\alpha /2}+Z_{1-\beta }\right)}^{2}}{\mu_{d}^{2}}$$

$$S_{p}^{2}=\frac{S_{1}^{2}{+S}_{2}^{2}}{2}$$

Based on the findings of Hegde et al. [24], at the 1% level of significance (Z_(1−α/2)_ = 2.58) and 80% power (Z_(1−β)_ = 0.84), with standard deviations of 1.54 (S1) in the device group and 2.14 (S2) in the control group, and a mean difference of 3.14 (µd), the sample size was estimated to be 9 per group. After adding a 20% attrition rate, the total sample size was increased to 11 participants per group (22 participants in total).

However, during the recruitment period, 38 eligible participants consented to take part and were included in this study, thereby increasing the precision of the study estimates.

### 2.3. Participant Selection

#### 2.3.1. Inclusion Criteria

Children aged 6–10 years who had a pre-treatment evaluation and were categorised as positive (+ or 3) or definitely positive (++ or 4) according to Wright’s modification of the Frankl Behaviour Rating Scale (FBRS), who underwent bilateral dental procedures requiring LA administration in the mandibular arches, and who exhibited overall physical and mental well-being and had no complicated medical history were included in the study.

#### 2.3.2. Exclusion Criteria

Children < 6 years of age; those with known allergies to LA, signs of irreversible pulpitis and dentoalveolar abscess; those with mental or physical disabilities; those categorised as negative (− or 2) or definitely negative (−− or 1), as determined using Wright’s modification of the FBRS; and those requiring LA in maxillary arches were excluded from the study.

### 2.4. Study Setting

The study involved 38 children, all of whom were outpatients at the clinic of the Department of Paediatric and Preventive Dentistry in Jazan.

#### 2.4.1. Test Intervention: Buzzy BEE^TM^

The intervention in this study was the use of an extraoral vibratory device, Buzzy BEE™ (Pain Care Labs, Atlanta, GA, USA), during IANB. The device was attached to a gel ice pack containing water, sodium polyacrylate and a mixture of isothiazolinones [22]. The gel pack was refrigerated before attaching to the device. The device was positioned at the mandibular ramus during IANB. The entire contents of the cartridge, containing Scandicaine 2% Speciale (mepivacaine hydrochloride and adrenaline), were administered using a conventional needle-syringe with a 27-gauge needle (HogenSpitze, C-K Dental Ind. Co., Ltd.; Bucheon, Gyeonggi, Republic of Korea). Participants were allowed to play with the Buzzy BEE prior to extraoral application, using a ‘tell, show, do’ technique to introduce them to the intervention.

#### 2.4.2. Standard Care

After topical anaesthesia was applied for 1 min, the children in the control group received the IANB using a conventional technique. The entire contents of the cartridge, containing Scandicaine 2% Speciale (mepivacaine hydrochloride and adrenaline), were administered using a conventional needle-syringe with a 27-gauge needle (HogenSpitze, C-K Dental Ind. Co., Ltd.; Bucheon, Gyeonggi, Republic of Korea).

### 2.5. Procedure [Figure 1—Flow Chart]

Using a computer-generated random sequence, the study randomly allocated 38 children—19 to each group—to two groups, namely Group A and Group B. Both groups received the test intervention at alternate visits.

During the first visit, Group A received standard care alone while Group B received the test intervention along with standard care. This step was followed by a 7-day washout period. During the second visit, participants in Group A received the test intervention along with standard care, whereas those in Group B received standard care alone. At each visit, each participant switched between the right and left sides of the allocation, receiving the intervention on one side and serving as the control on the other.

Once the patient was seated in a dental chair, a pulse oximeter was placed on the index finger to measure pulse rate, and the patient was asked to select a face from the Wong–Baker scale (WBS) to rate their pain after injection. Before initiating the trial, the required demographic details of every participant were recorded. The principal investigator was blinded and performed all the procedures.

### 2.6. Outcomes

One trained examiner measured the primary outcomes, such as pain, via subjective assessment using the WBS and via objective assessment using the Sound, Eyes and Motor (SEM) scale. Another trained examiner measured the secondary outcomes, such as pulse rate and FBRS. The parental scale was measured using parents’ opinions.

### 2.7. Primary Outcomes

#### 2.7.1. WBS

This scale is used for the subjective assessment of pain [25]. The scale comprises 6 facial expressions; each is assigned a numerical value between 0 and 10, representing the degree of pain.

#### 2.7.2. SEM

Using a mobile phone to record the video of participants before and after administering anaesthesia, their pain and behaviour were assessed using this objective scale. Parameters indicating comfort, mild discomfort, moderate pain and severe pain were used, and the total score ranged from 0 to 9. For each participant, pain was evaluated on a 0–3 scale for each component, namely sound, eyes and motor [26]. The score description is as follows:

Score 0 (Comfort): No sound of discomfort; no expression of pain in eyes; body appears Relaxed.
Score 1 (Mild discomfort): Non-specific sounds suggesting pain; wide eyes indicating concern without tears; hands indicating tension.
Score 2 (Moderately painful): Verbal complaint (raised voice); watery eyes; random movements of the body/arm, grimace and twitching.
Score 3 (Painful): Explicit verbal complaint; crying with tears running down the face; pulling away, punching and aggressive physical contact.

### 2.8. Parental Scale

Parents were asked to use this observational scale to evaluate the child’s tolerance. Tolerance was rated on a scale of 1–10, with higher numbers indicating greater tolerance, based on reactions such as zero tolerance, tolerance with crying, with tears only, with an expressionless face and with a smile [26]. The parents were asked to record these observations after the anaesthesia procedure, as an adjunctive objective scale to complement the other pain-perception scales used in this study. Although this scale has been used previously in paediatric dental research [26], detailed psychometric validation data on its reliability and validity are limited in the available literature.

### 2.9. Secondary Outcomes

#### 2.9.1. Pulse Rate

The pulse rate was measured using a pulse oximeter (Dr Trust Pulse Oximeter, Nureca Limited, India) to indicate anxiety levels. The device was affixed to the left index finger, and results were obtained in a relatively short time. ‘Before,’ ‘during’ and ‘after’ LA administration, pulse rate values were recorded. The ‘before’ values were obtained 15 min before administering LA to account for fluctuations, and the final mean was calculated. The average pulse rate readings were calculated as ‘during’ and ‘after’ values based on recordings made during the LA injection and 1 min after the injection.

#### 2.9.2. FBRS

The child’s behaviour before, during and after anaesthesia was evaluated using the FBRS. This 4-point ordinal scale assesses cooperation and response to evaluate the child’s behaviour throughout the dental procedure (1 = definitely negative, 2 = negative, 3 = positive, 4 = definitely positive) [27].

### 2.10. Statistical Analysis

SPSS 20 for Windows (Statistical Package for the Social Sciences, SPSS Inc., Chicago, IL, USA) was used to conduct statistical analyses. The chi-squared test was used to analyse participant distributions across factors such as gender, age and accompanying person, and the Shapiro–Wilk test was used to assess the normality of the data. Intergroup comparisons at each visit were conducted using the Mann–Whitney test for patient behaviour, parental scale and pain intensity, whereas the pulse rate between the two groups was analysed using the unpaired *t*-test.

To address the crossover design of this study, a crossover analysis was conducted to evaluate period, treatment and carryover effects. A linear mixed model was used to analyse continuous outcomes such as pulse rate, including treatment, sequence and period effects as fixed effects, with the participant as a random effect. For ordinal variables (WBS, FRBS and SEM), a non-parametric crossover analysis was conducted. The sum of responses across periods was compared using the Mann–Whitney test to assess the carryover effect. The difference between treatment and periods/phases was assessed using the Wilcoxon signed-rank test to evaluate treatment and period effects. A *p*-value of <0.05 was considered statistically significant.

## 3. Results

This study recruited 38 eligible participants, which exceeded the minimum required sample size of 22. Participants were randomly allocated to two treatment-sequence groups (conventional vs. Buzzy BEE technique), as shown in Table 1.

A statistically significant difference in pulse rate was observed during IANB at both the first (*p* = 0.02) and second (*p* = 0.002) visits, with a reportedly higher pulse rate in the group receiving conventional treatment (101.31 ± 5.68, 106.36 ± 7.61), as shown in Table 2.

Table 3 compares patient behaviour during IANB administration. It explains the FBRS for groups A and B during the first and second visits.

An extremely high, statistically significant difference in WBS scores was observed after IANB at the second visit (*p*-value < 0.001), with a higher median score of ‘four’ in Group B (conventional technique), as shown in Table 4.

SEM score analysis showed a significant difference for the ‘eyes’ component (*p* value = 0.004) across the groups during the second visit, with a median score of 1 in the group receiving the conventional technique. A statistically significant difference (*p* value = 0.002) was observed for the ‘motor’ component [0 (0, 0) 1 (0, 1)], and the Buzzy BEE group reported a reduced motor response during their second visit, as presented in Table 5.

The crossover analysis demonstrated a significant treatment effect on pulse rate (<0.001) but no significant effects on the other variables. No significant period effect was observed. Significant carryover effects were noted for WBS (<0.001) and SEM (0.004), as shown in Table 6.

## 4. Discussion

Children often consider needle pricks their most dreaded healthcare experience, causing severe pain, anxiety and distress and having a detrimental impact on both children and parents [28]. One of the most common intra-oral LA techniques is IANB, which is also considered one of the most painful and unpleasant procedures for children [29,30]. Hence, the most essential criterion for the successful treatment of paediatric patients is effective pain control [30]. Assessing the credibility of a chilled external vibration device, Buzzy BEE, for reducing pain perception in children during IANB was the primary goal of this study.

Pain in children is a multidimensional and complex phenomenon that depends on several attributes, such as location, quality of sensation and the child’s cognitive abilities [2]. Infants and young children communicate their intense experience of pain via behavioural and physiological indicators, such as irritability or placing a hand on the belly during abdominal pain [31]. As cognitive development is important for effective communication and self-reporting of pain, only children >6 years of age must be evaluated, the age at which cognitive development begins to manifest [2]. Many similar studies in the literature have recruited samples from this age range [2,19,32].

Assessing pain severity in children is challenging. While subjective evaluation of pain is considered the gold standard, the potential for children to exaggerate their pain cannot be overlooked [33]. Therefore, integrating a multidimensional pain assessment approach that incorporates children’s physiological responses, behavioural evaluation and self-report provides a more comprehensive understanding of the intervention’s effectiveness.

In this study, a significant reduction in pulse rate in the Buzzy BEE group during IANB was observed at both visits compared with the conventional group. Similarly, Shetty et al. reported a significant reduction in heart rate in children undergoing IANB in the Buzzy BEE group [22]. Other studies have also reported reductions in heart rate during various LA procedures when Buzzy BEE was used [15,21].

Elevated heart rate during dental procedures is associated with anxiety and stress. The failure rates of IANB have been documented to be higher in patients with dental anxiety [34]. The sympathetic nervous system—activated by anxiety and discomfort—releases glucocorticoids and catecholamines, which increase blood pressure, heart rate and oxygen saturation [35]. Thus, the observed reduction in pulse rate in the Buzzy BEE group may reflect reduced anxiety and improved physiological stability during the procedure [21]. However, Atik et al. did not observe any significant effect of Buzzy BEE on heart rate [32].

Contrary to the reduction in pulse rate observed in this study, no significant treatment effects were observed on the behavioural outcomes. Both objective and subjective pain perception were only significantly reduced at the second visit. The timing of the intervention did not influence behaviour as the analysis demonstrated no significant period effects. Hence, the behavioural difference on the second visit, as evidenced by the significant crossover effect identified in the analysis of WBS and SEM scores, can be attributed to familiarity with the environment and anticipated outcomes. Furthermore, the novelty and distractive characteristics of this vibrating device might have contributed to placebo-like effects, thereby influencing the subjective and behavioural responses observed during the second visit. Similarly, significant differences in SEM scores during local infiltration were only reported on the first visit in the prior study by Vishwanathaiah et al. [19]. The behavioural carryover effects could have led to the absence of significant differences on the subsequent visit [19]. The use of Buzzy BEE for IANB and other local anaesthetic procedures has been associated with significantly lower WBS scores in previous research [2,19], although a few studies did not report a significant decrease compared with their counterparts [32,33]. The study by Shetty et al. used the FLACC-R (face, legs, arms, cry and consolability revised) tool to assess children’s pain perception, demonstrating lower scores in children undergoing IANB while using Buzzy BEE [22]. A similarly significant decrease in pain perception, as indicated by FLACC scores, was reported among Turkish children [32]. However, a combination of pre-cooling and vibration using the Buzzy BEE did not decrease pain in Iranian children during the IANB injection, as reported by Narimany et al. [33].

A meta-analysis of three recent studies on extra-oral vibration devices did not demonstrate any statistically significant reduction in either objective or subjective pain perception, which may be attributed to factors such as the inclusion of only a few studies and the exclusion of other relevant studies because of rigorous eligibility criteria [30]. Moreover, children were found to be stressed by the devices’ vibration [36]. However, another meta-analysis evaluating the effect of vibratory devices on injection-related dental pain demonstrated that Buzzy BEE was more effective at reducing pain and fear than other vibration devices owing to its distractive features. The battery-operated, handheld ‘bee’-like Buzzy BEE has a colourful, animated design that helps distract paediatric patients from triggering stimuli, such as the needle [36]. In this study, the Buzzy BEE device was positioned at the mandibular ramus, thereby stimulating the underlying bone near the injection site and resulting in a greater reduction in pain [22].

Children with prior experience of injections may anticipate discomfort and pain, and these experiences can significantly shape their attitudes and emotional responses to subsequent injections [37]. Hence, adopting an appropriate distraction technique, such as Buzzy BEE, will be highly beneficial for ensuring successful treatment and follow-up [38]. Furthermore, clinicians’ competence and effective communication among the operator, nurse/assistant, parent and child are vital for optimal pain management and play a major role in the effective delivery of treatment [38]. One drawback of the device is its relatively large size compared with the smaller facial morphology of children [22]. However, as evidenced by the treatment effects on pulse rates, the Buzzy BEE effectively influences the physiological response of children undergoing IANB. Although behavioural responses to this device were influenced by carryover effects, it can still be implemented as a distraction strategy and as an adjunct to conventional behavioural management techniques from a clinical perspective.

The use of Buzzy BEE in various dental LA procedures has been studied, yet evidence of its effectiveness for the most challenging IANB remains limited. The study’s primary strength is its crossover design, which allowed each participant to serve as their own control, thereby reducing inter-individual variability. Furthermore, pain perception was comprehensively assessed. However, the split-mouth design can introduce psychological bias among participants, which may explain the discrepancies in statistical significance observed between visits. The study’s findings cannot be applied universally to the broader paediatric population as only cooperative children within a specific age group were included. Owing to the nature of the intervention, examiner blinding was not feasible, as the assessors directly observed the outcomes, which may have introduced observatory bias.

Furthermore, the parents were not blinded to the intervention, which might have also influenced their reporting of pain perception. Future studies should incorporate diverse age groups and adopt a multicentric design with appropriate parental blinding to validate the findings of this study. In addition, the cost-effectiveness of Buzzy BEE in a paediatric dental setting must be investigated.

## 5. Conclusions

In this study, the Buzzy BEE was found to effectively reduce pulse rates in children during IANB injections, suggesting a positive impact on anxiety levels. The improvements in subjective and pain perception observed with Buzzy BEE, noted only during the second visit, should be interpreted with due diligence. The significant carryover effects in this study limit the evidence for the role of Buzzy BEE in reducing pain perception during IANB injection. Hence, further studies with a larger sample size and extended washout periods are recommended to evaluate the effectiveness of pre-cooled external vibration devices in reducing pain perception during IANB.

## Figures and Tables

**Table 1:** Baseline demographic characteristics according to treatment sequence.

SI. No	Parameters	Group A N (%)	Group B N (%)	*p* Value
1	Age			1
	7–8 years	13 (68.4)	13 (68.4)	
	9–10 years	6 (31.5)	6(31.5)	
2	Sex			0.511
	Male	10 (52.6)	12 (63.2)	
	Female	9 (47.4)	7 (36.8)	

**Table 2:** Comparison of pulse rate during inferior alveolar block administration.

Visit	Parameter	Group A (Mean ± SD)	Group B (Mean ± SD)	*p* Value
First visit	Pulse rate Before anaesthesia	95.48 ± 9.19	96.01 ± 10.6	0.652
	During anaesthesia	101.31 ± 5.68	95.52 ± 9.23	0.02 *
	1 min after LA administration	99.68 ± 6.23	95.21 ± 7.47	0.527
Second Visit	Pulse rate Before anaesthesia	100.4 (92.8, 105.9)	96.2 (91.2, 102)	0.397
	During anaesthesia	98.47 ± 7.5	106.36 ± 7.61	0.002 **
	1 min after LA administration	97.63 ± 7.79	101.94 ± 8.09	0.103

* Significant at 0.05 level, ** Significant at 0.01 level.

**Table 3:** Comparison of patient behaviour during inferior alveolar block administration.

Visit	Parameters	Group A [Median (Q1, Q3)]	Group B [Median (Q1, Q3)]	*p* Value
First	FBRS Before anaesthesia	4 (3, 4)	4 (3, 4)	0.334
	During anaesthesia	3 (2, 3)	3 (2, 3)	0.456
	After anaesthesia	3 (3, 3)	3 (3, 4)	
Second	FBRS Before anaesthesia	4 (3, 4)	4 (3, 4)	1
	During anaesthesia	3 (2, 3)	2 (2, 3)	0.159
	After anaesthesia	3 (3, 3)	3 (3, 3)	1

FBRS = Frankl Behaviour Rating Scale.

**Table 4:** Comparison of the intensity of pain during inferior alveolar block administration.

Visit	Parameters	Group A [Median (Q1, Q3)]	Group B [Median (Q1, Q3)]	*p* Value
First	WBS			
	Before the procedure	0 (0, 0)	0 (0, 0)	NA
	After the procedure	4 (2, 6)	2 (2, 4)	0.071
Second	WBS			NA
	Before the procedure	0 (0, 0)	0 (0, 0)	
	After the procedure	2 (2, 3)	4 (3, 6)	0.001 ***

WBS = Wong–Baker Scale, * Significant at 0.05 level, ** Significant at 0.01 level, *** Significant at 0.001 level, NA—Not Applicable.

**Table 5:** Comparison of the intensity of pain during inferior alveolar block administration.

Visit	Parameters	Group A [Median (Q1, Q3)]	Group B [Median (Q1, Q3)]	*p* Value
First	Parental scale	8 (5.5, 9)	8 (6, 10)	0.238
Second	Parental scale	9 (6.5, 9)	7 (6, 8.5)	0.212
First	SEM			
	Sound	0 (0, 1)	0 (0, 1.5)	0.551
	Eyes	1 (1, 2)	1 (0, 1)	0.054
	Motor	1 (0, 1.5)	0 (0, 1)	0.074
Second	SEM			
	Sound	0 (0, 1.5)	1 (0.5, 2)	0.05
	Eyes	0 (0, 1)	1 (1.2)	0.004 **
	Motor	0 (0, 0)	1 (0, 1)	0.002 **

* Significant at 0.05 level, ** Significant at 0.01 level, *** Significant at 0.001 level.

**Table 6:** Crossover analysis depicting carryover, period and treatment effects.

Variables	Carryover Effect *p* Value	Period Effect *p* Value	Treatment Effect *p* Value
WBS	<0.001 **	0.295	0.224
SEM	0.004 *	0.917	0.474
FRBS	0.456	0.311	0.322
Pulse rate	0.508	0.10	<0.001 **

WBS = Wong–Baker Scale, SEM = Sound, eye and motor, FRBS = Frankl Behaviour Rating Scale, * Significant at 0.05 level, ** Significant at 0.01 level.

## Data Availability

The data presented in this study are available on request from the corresponding author. The data are not publicly available due to privacy and ethical reasons.

## Abbreviations

The following abbreviations are used in this manuscript:

FBRS	Frankl Behaviour Rating Scale
WBS	Wong–Baker Scale
SEM	Sound, Eyes and Motor Scale
